# PPARγ Regulates Triclosan Induced Placental Dysfunction

**DOI:** 10.3390/cells11010086

**Published:** 2021-12-28

**Authors:** Jing Li, Xiaojie Quan, Yue Zhang, Ting Yu, Saifei Lei, Zhenyao Huang, Qi Wang, Weiyi Song, Xinxin Yang, Pengfei Xu

**Affiliations:** 1 School of Public Health, Xuzhou Medical University, 209 Tong-Shan Road, Xuzhou 221002, China; quanxiaojie7@gmail.com (X.Q.); Feb_20th@163.com (Y.Z.); yuting20211213@163.com (T.Y.); huangzhenyao@gmail.com (Z.H.); wangqixzmu2018@163.com (Q.W.); songweiyi798@xzhmu.edu.cn (W.S.); xxyang@xzhmu.edu.cn (X.Y.); 2 Key Laboratory of Human Genetics and Environmental Medicine, Xuzhou Medical University, Xuzhou 221004, China; 3 Center for Pharmacogenetics and Department of Pharmaceutical Sciences, University of Pittsburgh, Pittsburgh, PA 15261, USA; sal208@pitt.edu; 4 Beijing Key Laboratory of Gene Resource and Molecular Development, College of Life Sciences, Beijing Normal University, Beijing 100875, China

**Keywords:** triclosan, PPARγ, placenta toxicity, cell migration, angiogenesis, inflammation

## Abstract

Exposure to the antibacterial agent triclosan (TCS) is associated with abnormal placenta growth and fetal development during pregnancy. Peroxisome proliferator-activated receptor γ (PPARγ) is crucial in placenta development. However, the mechanism of PPARγ in placenta injury induced by TCS remains unknown. Herein, we demonstrated that PPARγ worked as a protector against TCS-induced toxicity. TCS inhibited cell viability, migration, and angiogenesis dose-dependently in HTR-8/SVneo and JEG-3 cells. Furthermore, TCS downregulated expression of PPARγ and its downstream viability, migration, angiogenesis-related genes *HMOX1*, *ANGPTL4*, *VEGFA*, *MMP-2*, *MMP-9*, and upregulated inflammatory genes *p65*, *IL-6*, *IL-1β*, and *TNF-α* in vitro and in vivo. Further investigation showed that overexpression or activation (rosiglitazone) alleviated cell viability, migration, angiogenesis inhibition, and inflammatory response caused by TCS, while knockdown or inhibition (GW9662) of PPARγ had the opposite effect. Moreover, TCS caused placenta dysfunction characterized by the significant decrease in weight and size of the placenta and fetus, while PPARγ agonist rosiglitazone alleviated this damage in mice. Taken together, our results illustrated that TCS-induced placenta dysfunction, which was mediated by the PPARγ pathway. Our findings reveal that activation of PPARγ might be a promising strategy against the adverse effects of TCS exposure on the placenta and fetus.

## 1. Introduction

Triclosan (TCS) is a synthetic broad-spectrum antimicrobial and exposure mainly occurs in dermal application (soaps, hand sanitizers, toothpaste, cosmetics, antiperspirants, and bedclothes) and oral use of consumer products (water, food products) [1,2,3]. TCS, as a kind of exogenous biological signal termed Endocrine disruptors (EDCs), can mimic endogenous estrogenic hormones, and interferes with the maintenance of homeostasis and the regulation of developmental processes [4]. Previous epidemiological research demonstrated that TCS has been distinguished in mothers’ milk (1–13.6 ng/mL), urine (2.5–107 ng/mL), and cord blood samples [5,6,7]. In addition, high urinary TCS levels in patients with spontaneous abortion have been reported [8]. A Denmark Odense Child Cohort study stated that median unadjusted urinary TCS was 0.88 ng/mL in pregnant women, and high maternal urinary TCS levels were associated with reduced head and abdominal circumference at birth [9]. Animal experiments suggested that the exposure of pregnant mice to TCS reduced fetal body weight and viability [8]. TCS has been detected with a concentration of up to 478 ng/L and 1329 ng/g in surface waters and sediment, respectively, in China rivers [10]. Therefore, a further understanding of the toxicity of TCS is important for the inhibition of TCS pollution.

The nuclear receptors, peroxisome proliferator-activated receptors (PPARs) are activated by binding natural ligand-inducible transcription factors such as glitazones [11,12]. PPARs are responsible for metabolism and cell function and have three subtypes including PPARα, PPARβ/δ, and PPARγ [13,14]. PPARγ is highly expressed in reproductive tissues (ovary, testis, uterus, prostate, mammary gland) and the trophoblast labyrinthine zone in rodent placentas and human placentas [15]. PPAR has been suggested to be involved in regulating cell trophoblast proliferation, inflammatory reactions oxidative response, and nutrient transport to mediate placenta development [16,17,18]. Additionally, research has illustrated that PPARγ agonists enhanced, while PPARγ antagonists reduced, proliferative and migratory capabilities of endothelial cells [19]. The vasculature defects were observed in placentas at embryonic GD 9.5 in PPARγ-null mice [16]. Similar animal experiments implicated embryonic lethality induced by defects in placental vascularization in PPARγ-null mice [20,21]. The downregulated protein level of PPARγ was associated with placental disorders [22]. PPARγ expression levels were also found to be decreased in pregnant mice or zebrafish when exposed to TCS [23,24].

The effect of prenatal TCS exposure on placental toxicity and its role in fetal growth and development are still unclear. Whether the effect of prenatal TCS exposure on placental toxicity is associated with PPARγ remains to be revealed. Our research aims to clarify the potential character of prenatal TCS exposure on placenta function and its potential mechanism of PPARγ in the process of placental exposure to TCS.

## 2. Materials and Methods

### 2.1. Reagents

TCS (CAS No. 3380-34-5, purity > 98% pure) was obtained from Sigma–Aldrich (Oakville, ON, Canada). DMEM/F12 medium and MEM medium were purchased from KeyGEN BioTECH (Jiangsu, China). RPMI 1640 medium was purchased from GIBCO (Grand Island, N.Y.). Dimethyl sulfoxide (DMSO), fetal bovine serum (FBS), the Cell Counting Kit-8 (CCK-8), and corn oil used was obtained from Vicmed (Busan, Korea) and Macklin (Shanghai, China), respectively, and were analytical grades. The pcDNA-*PPARγ* vector and *si-PPARγ* were purchased from GenePharma (Shanghai, China). Moreover, the rosiglitazone (a specific PPARγ agonist) and GW9662 (a specific PPARγ antagonist) were obtained from MedChemExpress (Shanghai, China).

### 2.2. Cell Culture and Transfection

Thanks to Dr. Xinru Wang from Nanjing Medical University (Nanjing, China) for sending HTR-8/SVneo and JEG-3 cells as a gift to us. HTR-8/SVneo and JEG-3 cells were seeded in DMEM/F12 and MEM medium, respectively, and supplemented with 10% FBS in a 5% CO_2_ humidified atmosphere at 37 °C. Based on the manufacturer’s instructions, these two cell lines were transfected with small interfering RNA (siRNA) (*si-PPARγ*; 20 nM), the control siRNA (*si-Con*; 20 nM), pcDNA-*PPARγ* (2 µg), and pcDNA 3.1 (2 µg) targeting PPARγ by Lipofectamine 2000 (Invitrogen, Carlsbad, CA, USA). The medium was replaced after 4 h of cell transfection. After cell transfection for 24 h, they were incubated with triclosan for 24 h. For PPARγ activation or deactivation, cells were treated with rosiglitazone or GW9662 with 10 µM for 24 h and pre-treated for 0.5 h before incubating with TCS.

### 2.3. Animal Treatment

Institute of Cancer Research (ICR) mice, at 10 weeks old and weighing about 30 g, were obtained under a protocol approved by Xuzhou Medical University Animal Center. On day GD 7.5, after the vaginal plug (GD 0.5) was observed in pregnant mice, they were randomly separated into five groups (*n* = 8 per group). All pregnant mice were orally managed with 0, 10, 50, and 100 mg/kg/day TCS and from GD 7.5 to GD 17.5, while control pregnant mice accepted the same volume of corn oil. Firstly, rosiglitazone was dissolved in DMSO, and the ratio of DMSO to corn oil was 1:1000, and pregnant mice were orally administered with rosiglitazone (20 mg/kg/day) together with TCS (100 mg/kg/day). Mice placentas were isolated, put into liquid nitrogen to be quickly frozen and stored at −80 °C. According to manufacturers of Xuzhou Medical University Animal Ethics Committee, the entire experiment was performed in a Specified Pathogen-Free (SPF) environment (protocols 201605w025, 25 May 2016 and 202106A237, 25 June 2021).

### 2.4. Cell Viability Assay

HTR-8/SVneo or JEG-3 cells (5 × 10^3^ cells/well) were seeded into sterile plates of 96 wells in complete medium (DMEM/F12, MEM, 10% FBS). Cells were incubated and cultured with TCS of different concentrations for 24 h. An amount of 10 μL CCK-8 (Vicmed, Busan, Korea) solution was added to each well and incubated for 0.5–4 h at 37 °C in a 5% CO_2_ atmosphere. The plate was detected at 450 nm on a microplate reader Spark (TECAN, Austria, 30086376).

### 2.5. Cell Migration Assay

HTR-8/SVneo or JEG-3 cells were grown in 6-well cell plates overnight to obtain approximately 70% confluency. After corresponding treatment, HTR-8/SVneo and JEG-3 cells were starved in 1% serum medium. Scratch wounds were made via a sterile 10 μL pipette tip to obtain three parallel lines. Multiple photographs were taken of the wound using an inverted microscope (KS400; Carl Zeiss Imaging GmbH), and the migration distance was measured by Image J analysis software (National Institutes of Health, v1.8.0). The whole wound closure distance was calculated and the distance of newly covered cells was measured at 0 and 24 h to evaluate migration.

### 2.6. Tube Formation Assay

Approximately 50 µL Matrigel (10 mg/mL) (BD Biosciences, San Diego, CA, USA) was decked into 96-well plates and allowed to completely solidify at 37 °C for 1 h to form a gel. Approximately 5 × 10^3^/mL HUVECs were suspended in a conditioned medium (the conditioned medium was derived from HTR-8/SVneo and JEG-3 cells and added into the wells of the Matrigel solidified plate. Tube formation in each well was monitored and imaged using an inverted microscope after the plate was incubated at 37 °C for 5 h. We repeated the experiment three times independently. Tube branch length was measured with six duplicates per well, while average branch length was taken from three random microscopic fields per well. Each assay was done in triplicate and quantification was conducted with Image J software (National Institutes of Health, v1.8.0).

### 2.7. Real Time PCR (RT-PCR)

Placental tissues, trophoblast cells, and Trizol (Vicmed, Busan, Korea) were used to isolate total RNA. RNA concentration was detected by Nanodrop 2000 (Thermo, Scientific). The reverse transcription was conducted using SYBR Green qPCR SuperMix and 500 ng of the total RNA was reverse transcribed to cDNA. The quantitative RT-PCR was carried out using SYBR Green Master Mix on a 7500 fast real-time PCR System (Applied Biosystem, Foster, CA, USA) according to the manufacturer’s instructions. The mRNA levels were analyzed using the comparative cycle threshold method (2^−ΔΔCT^), and the relative levels were normalized to the level of GAPDH. The primers were designed by Sangon Biotech (Shanghai, China), and primer sequences were listed in Supplementary Table S1.

### 2.8. Western Blot Analysis

Total cellular protein was extracted and lysed with RIPA lysis buffer with protease inhibitors (KeyGEN, Nanjing, China) and phosphatase inhibitors (KeyGEN, Nanjing, China). Proteins were separated with 10% SDS-PAGE gels and transferred to PVDF membranes (Merck Millipore, MA, USA). Membranes were blocked with 5% non-fat milk for 1h and then incubated with primary antibodies of PPARγ, ANGPTL4, MMP-2, IL-1β, GAPDH, and tubulin β (Bioword, Beijing, China) overnight at 4 °C. After being washed five times for five minutes, the membranes were incubated with the corresponding secondary antibodies for 1h at room temperature. The protein bands were visualized with an Enhanced Chemiluminescence (ECL) detection kit (Amersham, NJ, USA). The protein bands were analyzed using Image Lab Software (Bio-Rad, CA, USA).

### 2.9. Statistical Analysis

The above assays were carried out three independent times and all data were carried out by GraphPad Prism 8.3 software (San Diego, CA, USA), and data were exhibited as the mean ± SEM. The comparison of the data was subjected to a one-way analysis of variance among each independent group. The *t*-test or one-way ANOVA was implemented using the SPSS 19.0 (IBM, Armonk, NY, USA). *p*-value < 0.05 and *p*-value < 0.01 were considered a statistically significant difference and higher significance, respectively.

## 3. Results

### 3.1. PPARγ Is Crucial in Cell Viability Inhibition Induced by TCS

**Figure 1:**
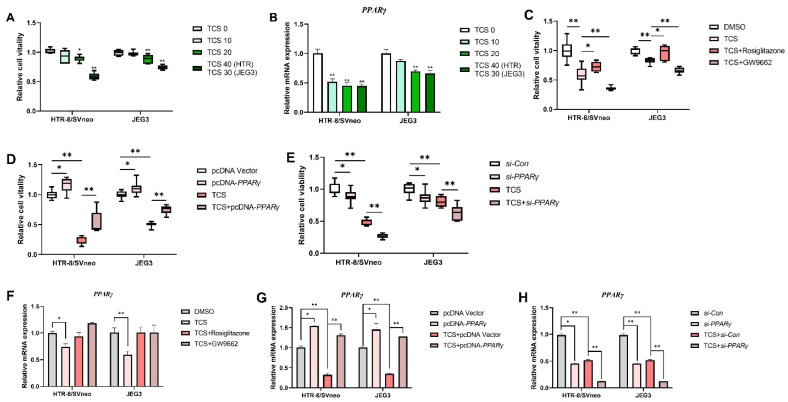
The viability of TCS on HTR-8/SVneo and JEG-3 cells was detected by Cell Counting Kit-8 assay for 24 h. (**A**) Cell vitality in HTR-8/SVneo and JEG-3 cells exposed to TCS. (**B**) Expression of PPARγ was determined by RT-PCR in HTR-8/SVneo and JEG-3 cells exposed to indicated TCS for 24 h. (**C**) Cell vitality was detected when exposed to TCS (40 µM for HTR-8/SVneo, 30 µM for JEG-3) in the absence or presence of rosiglitazone or GW9662 in the two cell lines. PPARγ was overexpressed (**D**) and knockdown (**E**) in the two cell lines and co-treated with TCS in HTR-8/SVneo and JEG-3 cells while cell vitality was analyzed. (**F**) HTR-8/SVneo and JEG-3 cells exposed to TCS while in the absence or presence of rosiglitazone (10 µM) and GW9662 (10 µM). HTR-8/SVneo and JEG-3 cells exposed to TCS during PPARγ overexpression (**G**) or knockdown (**H**). The data are shown as the means ± S.E.M. * *p* < 0.05; ** *p* < 0.01; compared with the indicated group, *n* = 3.

The cell viability of HTR-8/SVneo and JEG-3 cells exposed to TCS at different concentrations was determined by the CCK-8 assay. TCS dose-dependently inhibited cell viability of HTR-8/SVneo and JEG-3 cells, significantly decreasing cell viability at 20 μM, 30 μM, or 40 μM (Figure 1A). Of interest, TCS significantly inhibited *PPARγ* mRNA expression levels in those two cell lines especially HTR-8/SVneo cells (Figure 1B).

To investigate whether PPARγ was involved in the TCS-induced inhibition of cell viability, cell viability was analyzed when PPARγ was overexpressed and activation (rosiglitazone) or knockdown and inhibition (GW9662), and then exposed to TCS. Rosiglitazone and GW9662 with the concentration of 10 μM were not toxic in HTR-8/SVneo and JEG-3 cells (Figure S1A). The efficiency of PPARγ overexpression and knockdown is shown in Figure S1B–D. PPARγ mRNA and protein expression level was increased when PPARγ was overexpressed (Figure S1B,C) but decreased after knockdown compared to the control in HTR-8/SVneo and JEG-3 cells (Figure S1B,D). The results exhibited that pcDNA-*PPARγ* and rosiglitazone alleviated TCS-elicited cell viability inhibition in HTR-8/SVneo and JEG-3 cells (Figure 1C,D). In contrast, GW9662 and *si-PPARγ* aggravated the inhibition in these two cell lines (Figure 1C,E). In addition, treatment with rosiglitazone increased the expression of PPARγ-regulated genes, such as *HMOX1*, *ANGPTL4*, *VEGFA*, *MMP-2*, and *MMP-9*, and decreased the expression of *p65*, *IL-6*, *IL-1β*, and *TNF-α* in vitro. On the other hand, treatment with GW9662 has an opposite trend (Figure S1E).

### 3.2. PPARγ Is Involved in TCS-Elicited Impaired Migration

**Figure 2:**
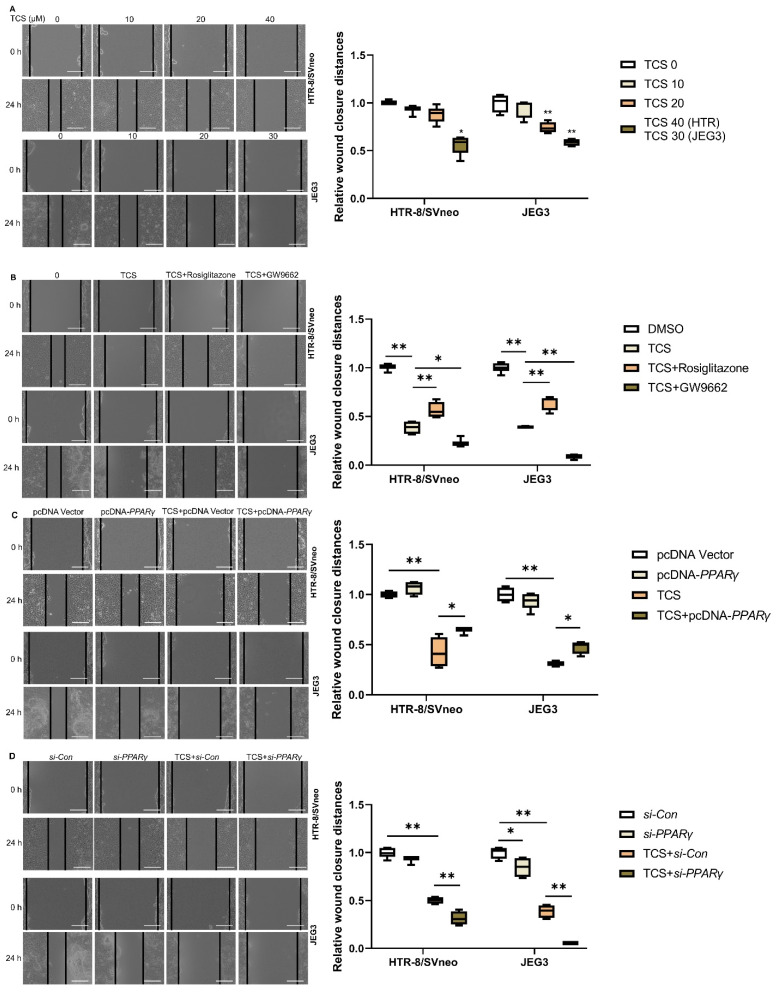
Role of PPARγ in migration exposed to TCS in vitro. The migration distances were measured after exposure to TCS at 0 h and 24 h. (**A**) HTR-8/SVneo and JEG-3 cell migration distance was decreased with increasing concentration of TCS. Co-treated with TCS in the absence or presence of rosiglitazone or GW9662 in HTR-8/SVneo and JEG-3 cells (**B**). The migration distance in response to PPAR overexpression (**C**) and knockdown (**D**) when exposed to TCS. Scale bar: 200 µm. The relative wound closure distances are shown on the right. The data are shown as the means ± S.E.M. * *p* < 0.05; ** *p* < 0.01; compared with the indicated group, *n* = 3.

The wound healing assay confirmed that the migration ability of HTR-8/SVneo and JEG-3 cells were inhibited after concentration dependent on exposure to TCS (Figure 2A). To assess the impact of TCS on cell migration influenced by PPARγ in vitro, the migration assays were conducted in the absence or presence of rosiglitazone or GW9662. Our results in Figure 2B demonstrated that treatment with rosiglitazone significantly increased cell migration, while GW9662 decreased the cell migration compared to the TCS-exposed group in HTR-8/SVneo and JEG-3 cells. PPARγ displayed overexpression and knockdown in HTR-8/SVneo and JEG-3 cells. Our results indicated that PPARγ overexpression alleviated the migration inhibition induced by TCS in HTR-8/SVneo and JEG-3 cells (Figure 2C). Moreover, PPARγ knockdown aggravated the migration inhibition induced by TCS in these two cell lines (Figure 2D).

### 3.3. PPARγ Alleviates TCS-Elicited Angiogenesis Inhibition

**Figure 3:**
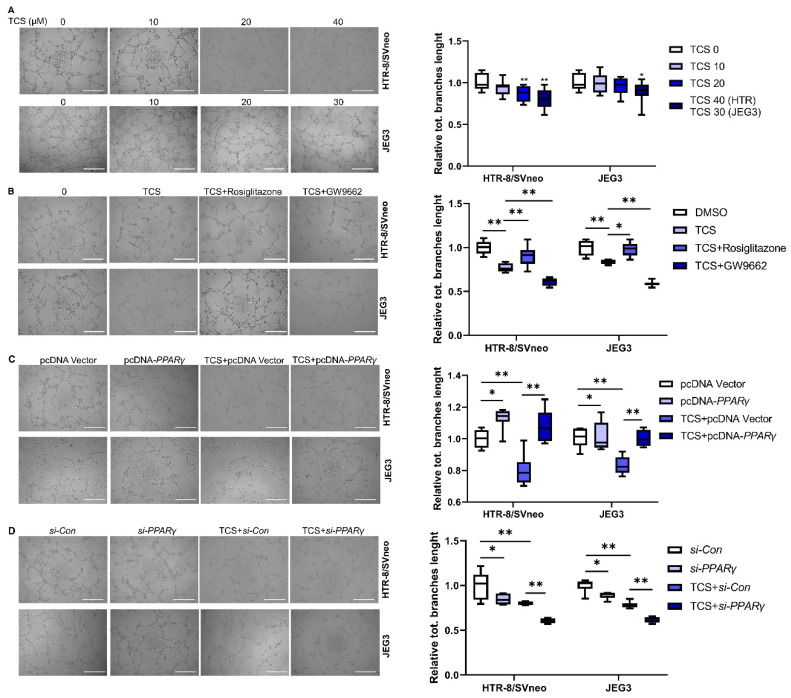
TCS reduced HTR-8/SVneo and JEG-3 cells angiogenesis through PPARγ pathway. (**A**) TCS decreased the branch length of HTR-8/SVneo and JEG-3 cells. (**B**) Co-treated with TCS (40 µM for HTR-8/SVneo, 30 µM for JEG-3) in the absence or presence of rosiglitazone and GW9662 in these two cells. (**C**,**D**) PPARγ overexpression alleviated, while (**C**) PPARγ knockdown exacerbated. (**D**) TCS-induced cell angiogenesis inhibition of HTR-8/SVneo and JEG-3 cells. Scale bar: 400 µm. The data are shown as the means ± S.E.M. * *p* < 0.05; ** *p* < 0.01; compared with the indicated group, *n* = 3.

An angiogenesis assay was implemented to describe the influence of TCS on tube branch length. The HTR8/SVneo and JEG-3 cell lines were treated with different concentrations of TCS. TCS showed significantly decreased tube branch length compared to the control (DMSO) at 30 μM or 40 μM in the two cell lines, respectively (Figure 3A). The impact of PPARγ on TCS-elicited tube branch length is displayed in Figure 3. The existing results illustrated that PPARγ overexpression or rosiglitazone co-treatment alleviated the inhibition of tube branch length induced by TCS in vitro (Figure 3B,C). In contrast, PPARγ knockdown or GW9662 co-treatment aggravated the inhibition of tube branch length induced by TCS in HTR8/SVneo and JEG-3 cells (Figure 3B,D).

### 3.4. TCS Alters the Expression of PPARγ Target Genes Associated with Viability, Angiogenesis, and Migration

**Figure 4:**
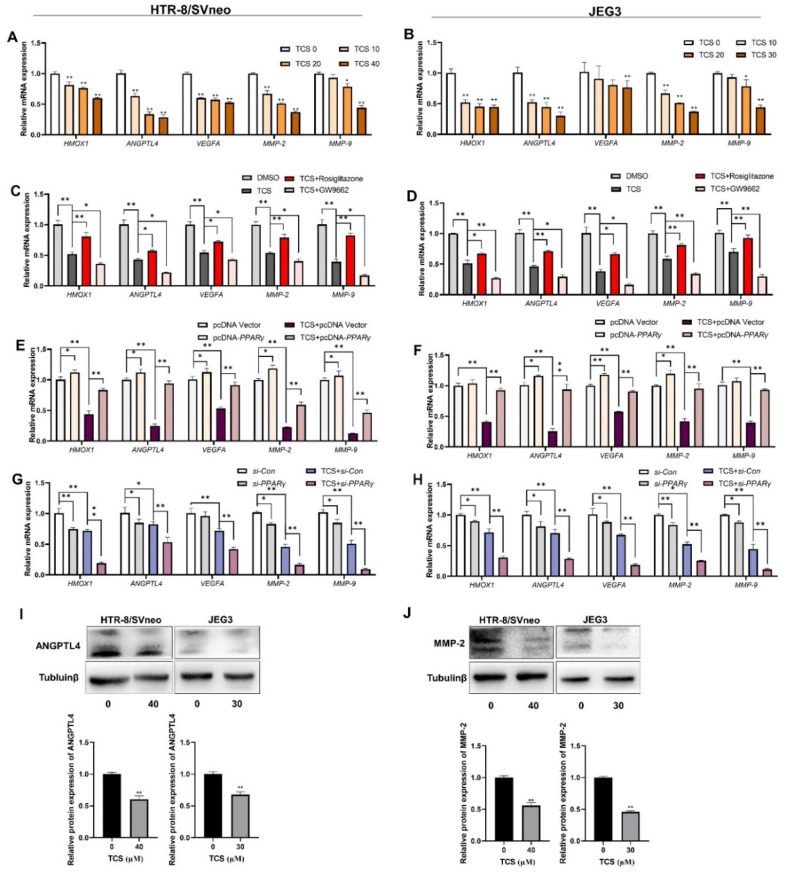
TCS inhibited expression of PPARγ target genes related to cell vitality, migration, and angiogenesis via PPARγ pathway in vitro. (**A**,**B**) The mRNA expression was analyzed by RT-PCR exposed to the indicated dose of TCS in HTR-8/SVneo and JEG-3 cells. (**C**,**D**) HTR-8/SVneo and JEG-3 cells treated with or without rosiglitazone and GW9662 for 24 h in HTR-8/SVneo and JEG-3 cells exposed to TCS (40 µM for HTR-8/SVneo, 30 µM for JEG-3). (**E**–**H**) PPARγ was overexpressed (**E**,**F**) or displayed knockdown (**G**,**H**) and co-treated with TCS in HTR-8/SVneo and JEG-3 cells. (**I**,**J**) The protein expression of ANGPTL4 (**I**) and MMP-2 (**J**) was analyzed by Western blot in HTR-8/SVneo and JEG-3 cells exposed to TCS. The relative values of protein expression are shown below. The data are shown as the means ± S.E.M. * *p* < 0.05; ** *p* < 0.01; compared with the indicated group, *n* = 3.

The *HMOX1*, *ANGPTL4*, *VEGFA*, *MMP-2*, and *MMP-9* genes were determined as the target genes of PPARγ and play considerable roles in cell viability, angiogenesis, and migration [25,26,27]. In comparison with the control group, the levels of *HMOX1*, *ANGPTL4*, *VEGFA, MMP-2,* and *MMP-9* mRNA were significantly decreased in the TCS-exposed group (Figure 4A,B). In addition, the protein levels of *ANGPTL4* and *MMP-2* were significantly decreased in the TCS-exposed groups (Figure 4I,J). We also assessed the influence of *PPARγ* on the above genes in the absence or presence of TCS. The results revealed that rosiglitazone enhanced the expression of *HMOX1*, *ANGPTL4*, *VEGFA*, *MMP-2*, and *MMP-9* levels, although TCS decreased them in vitro (Figure 4C,D). The *HMOX1*, *ANGPTL4*, *VEGFA*, *MMP-2*, and *MMP-9* mRNA levels were upregulated after overexpression of PPARγ in vitro (Figure 4E,F). In contrast, PPARγ knockdown downregulated the cell viability, angiogenesis, and migration-related genes in HTR-8/SVneo and JEG-3 cells (Figure 4G,H). Therefore, TCS inhibited the PPARγ pathway, leading to the downregulation of *HMOX1*, *ANGPTL4*, *VEGFA*, *MMP-2*, and *MMP-9.*

### 3.5. TCS Changes Expression Level of PPARγ-Regulated Inflammation Genes

**Figure 5:**
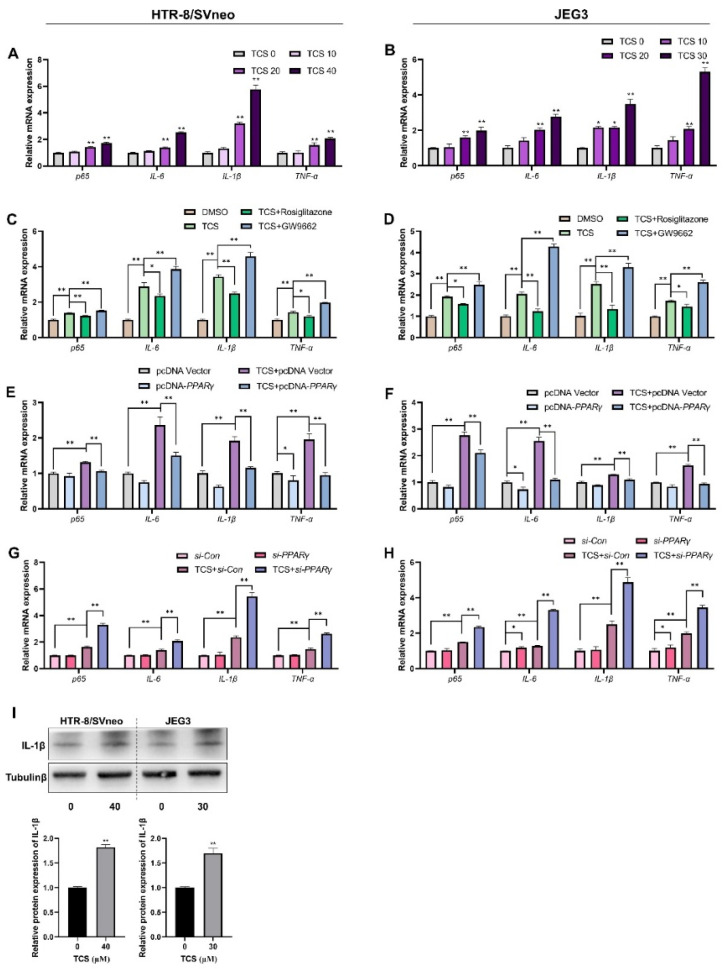
TCS increased expression of PPARγ-regulated genes related to inflammation through PPARγ pathway in vitro. (**A**,**B**) The mRNA expression level of the inflammatory genes exposed to indicated dose of TCS in HTR-8/SVneo and JEG-3 cells. (**C**,**D**) HTR-8/SVneo and JEG-3 cells treated with or without rosiglitazone and GW9662 for 24 h in HTR-8/SVneo and JEG-3 cells co-treated with TCS (40 µM for HTR-8/SVneo, 30 µM for JEG-3). (**E**–**H**) PPARγ was overexpressed (**E**,**F**) or displayed knockdown (**G**,**H**) and co-treated with TCS in HTR-8/SVneo and JEG-3 cells. (**I**) The protein expression of IL-1β was analyzed by Western blot in HTR-8/SVneo and JEG-3 cells exposed to TCS. The relative value of protein expression is shown below. The data are shown as the means ± S.E.M. * *p* < 0.05; ** *p* < 0.01; compared with the indicated group, *n* = 3.

The effect of TCS on PPARγ-regulated inflammatory genes was investigated, and results demonstrated that TCS was able to upregulate genes involved in inflammation such as *p65*, *IL-6*, *IL-1β*, and *TNF-α* in HTR-8/SVneo and JEG-3 cells (Figure 5A,B). The protein levels of *IL-1β* were significantly increased in TCS-exposed groups (Figure 4I). However, PPARγ overexpression or rosiglitazone co-treatment significantly alleviated the level of these inflammatory cytokines elicited by TCS in vitro (Figure 5C–F). In addition, PPARγ knockdown or GW9662 co-treatment enhanced the expression levels of those genes elicited by TCS in HTR-8/SVneo and JEG-3 cells (Figure 5C–H). These results emphasized that TCS upregulated inflammatory gene expression through the PPARγ pathway.

### 3.6. TCS Induces Placenta Dysfunction through PPARγ Pathway in Mice

**Figure 6:**
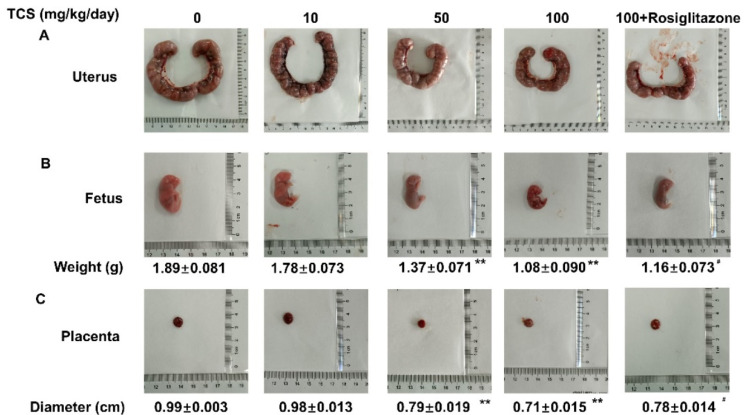
PPARγ participated in placental and fetal development toxicity of TCS. Representative picture of uterus (**A**), fetus (**B**), and placenta (**C**) of GD17.5 mice exposed to indicated doses of TCS. The data are shown as the means ± S.E.M; * *p* < 0.05 and ** *p* < 0.01, compared with control group; ^#^ *p* < 0.05, compared with TCS (100 mg/kg/day) group; *n* = 8.

To reveal the potential toxicity of TCS on the placenta and fetal development, pregnant ICR mice were treated with TCS daily by gavage at doses of 0, 10, 50, and 100 mg/kg/day from gestation day GD7.5 to GD17.5. Uterus, placenta, and fetus were collected for analysis in our study. As the results showed, the size of uterus (Figure 6A), fetus weight (Figure 6B), and placenta (Figure 6C) in TCS-exposed mice were decreased obviously in the 50 and 100 mg/kg/day gavage group compared to the vehicle group, indicating the serious placenta toxicity of TCS. Moreover, rosiglitazone (20 mg/kg/day) administration prevented the decrease of fetus weight (Figure 6B) and placenta diameter (Figure 6C) induced by TCS.

### 3.7. TCS Alters Expression of PPARγ-Regulated Genes in Mice Placenta

**Figure 7:**
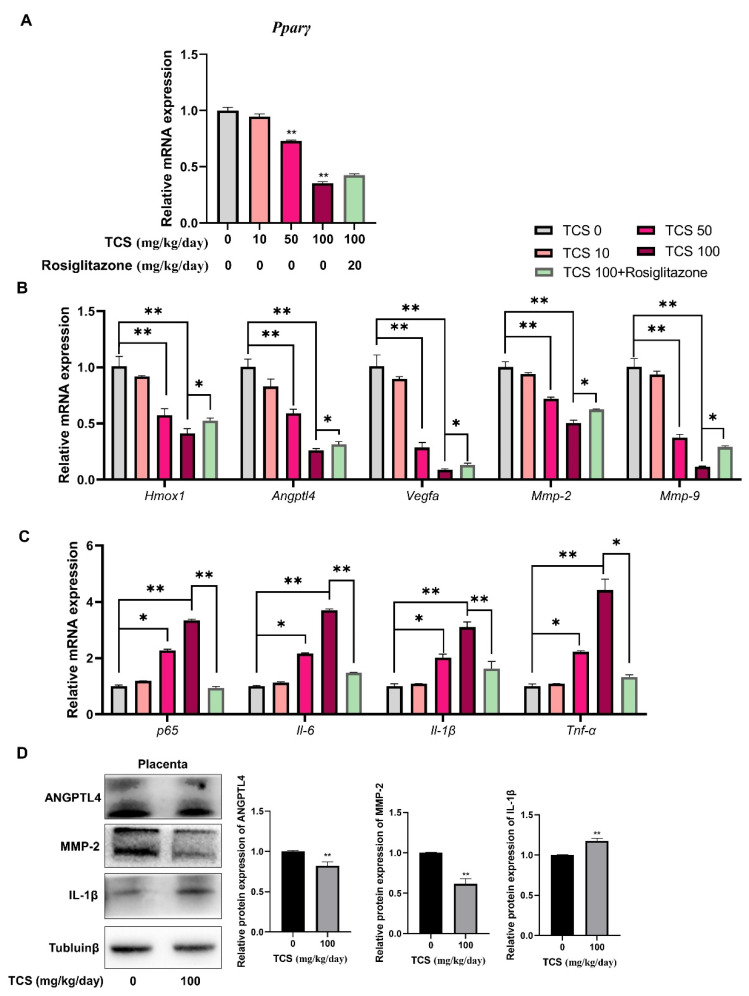
The gene and protein expression of PPARγ-regulated genes in placentas of gestational mice exposed to TCS. (**A**) The PPARγ expression level was detected by RT-PCR in GD17.5 mice placentas. (**B**,**C**) The mRNA expression of cell growth, angiogenesis, migration (**B**), and inflammation (**C**)-related genes in the placenta of GD17.5 mice treated with indicated doses of TCS and with or without rosiglitazone. (**D**) The protein expression of ANGPTL4, MMP-2, and IL-1β were analyzed by Western blot in placenta of GD17.5 mice treated with or without TCS (100 mg/kg/day). The relative values of protein expression are shown on the right. The data are shown as the means ± S.E.M. * *p* < 0.05; ** *p* < 0.01; compared with the indicated group, *n* = 8.

The effects of gestational TCS exposure on viability, migration, angiogenesis, and inflammatory genes in mice placenta were analyzed. As described in Figure 7, placental *Pparγ* (Figure 7A) and *Pparγ*-regulated genes *Homx1*, *Angptl4*, *Vegfa*, *Mmp-2*, and *Mmp-9* mRNA levels (Figure 7B) were decreased, while inflammatory genes *p65*, *Il-6*, *Il-1β*, and *Tnf-α* mRNA levels (Figure 7C) were significantly elevated in TCS-exposed pregnant mice. The protein expression of ANGPTL4, MMP-2, and IL-1β were consistent with the gene expression trends in the placenta of GD17.5 mice treated with or without TCS (Figure 7D). In addition, treatment with rosiglitazone reversed the PPARγ-regulated viability, migration, angiogenesis, and inflammatory gene expression induced by TCS (Figure 7B,C).

## 4. Discussion

In spite of numerous developmental toxicities shown to be triggered by TCS, the mechanism of TCS-elicited severe placental dysfunction has not been well elaborated. Here, our research revealed that TCS dose-dependently inhibited cell growth, migration, angiogenesis in HTR-8/SVneo and JEG-3 cells, and impaired placental development in mice. The mechanism of TCS-induced effects was studied. PPARγ was partly involved in the toxicity of TCS by regulating placental cell growth, migration, angiogenesis, and inflammatory responses in vitro and in vivo.

Abnormal placental cell proliferation may induce pregnancy complications such as fetal growth retardation, miscarriage, preeclampsia, and macrosomia [28]. Previous studies have verified that TCS had cytotoxic impression, elicited apoptosis, and inhibited cell vitality of human placental trophoblasts [29,30]. Placenta angiogenesis works for placental transport, endocrine, metabolic, and immune function regulation. It is essential for embryogenesis and is involved in the reproductive cycle and wound healing [16]. The intra-placental vascular lesions may result in preeclampsia, fetal growth restriction, or birthweight decrease [31]. Previous research has reported that TCS exposure decreased fetal viability and fetal body weight through placental thrombosis [32]. Some evidence has also demonstrated that TCS stimulated vascular branch disappearance and vascular injury in zebrafish [33]. In our animal research, placental diameter and fetal weight significantly decreased in pregnant mice in the TCS high-dose group. Previous studies have illustrated that the placenta had the greatest bio-accumulation of TCS, and reduced uterine weight and abortion were observed in the TCS (600 mg/kg/day) group in rats [34]. Following TCS exposure, reduction of gravid uterine weight and the occurrence of abortion was observed in pregnant rats [34]. PPARγ-deficient mice have placental abnormalities and defects in trophoblast differentiation and vascular development [20]. In addition, rosiglitazone increased placental vascularization and trophoblast migration and invasion in villous cytotrophoblast cells (VCT) and placental explants [35]. Our results also proved that PPARγ was inhibited in the mice placentas after treatment with TCS, and rosiglitazone prevented TCS-induced placenta toxicity by activation of PPARγ. It will be interesting to know the mechanism by which TCS affects PPARγ expression. Our results demonstrated that TCS inhibited cell growth, migration, and angiogenesis and influenced placenta development through the PPARγ pathway in vitro and in vivo.

Vascular endothelial growth factors A (*VEGFA*) and Heme oxygenase 1 (*HO-1*) are key modules of the angiogenesis process [36]. Angiopoietin-like protein 4 (*ANGPTL4*) is a secretory glycoprotein member of the angiopoietin family, which participates in the regulation of migration and angiogenesis [37,38]. Furthermore, functional studies found that PPARγ targeted *ANGPTL4* in placental development and angiogenesis, mediating the survival, proliferation, migration, and invasion of HTR8/SVneo cells [39,40]. Our results indicated that PPARγ activation alleviated the decrease of *HMOX1*, *ANGPTL4,* and *VEGFA* expression caused by TCS. MMPs have been demonstrated to mediate the migration and invasion of trophoblast cells, and *MMP-2* and *MMP-9* expression levels were decreased in preeclamptic placental tissues [41,42]. Similarly, our findings suggested that PPARγ mediated TCS-induced migration inhibition via *MMP-2* and *MMP-9* expression. In this study, cell growth, migration, and angiogenesis inhibition by TCS was partly ameliorated by PPARγ elevation or activation. These findings prove that TCS affects cell viability, migration, and angiogenesis through the PPARγ pathway.

Pregnancy is a process of dynamic inflammatory phases, and the inflammatory state is present almost throughout pregnancy and towards the end [43]. Activation of PPARγ is reported to suppress inflammation through NF-kappaB and TNF-α [44]. Indeed, pro-inflammatory conditions have been associated with pregnancy complications such as intrauterine growth restriction and preeclampsia [45]. TCS increased the inflammatory response by promoting *TNF-a* and *IL-6* expression in HUVEC [32]. Interestingly, PPARγ has a critical role in regulating inflammatory cytokines including *TNF-α* and *IL-6,* which were linked to preterm labor, miscarriage, and preeclampsia [22]. Moreover, PPAR-γ has been proved for its anti-inflammatory effects and downregulation of the expression of pro-inflammatory cytokines, such as *IL-6*, *IL-8*, and *TNF-α*, in human gestational tissue [46,47]. Furthermore, activation of PPARγ by rosiglitazone reversed the LPS-mediated effects on inflammatory cytokine release and proliferation inhibition in HTR-8/SVneo cells [48]. Similarly, we demonstrated that TCS can induce the expression of inflammatory genes in two cell lines. Rosiglitazone or PPARγ overexpression alleviated PFOS-induced cell growth, migration, angiogenesis inhibition, and the release of inflammatory cytokines in HTR-8/SVneo and JEG-3 cells [49]. Previous studies indicated that decreased PPARγ expression or the inhibition of PPARγ activity led to mitochondrial fission, hyperpolarization, and increased oxidative stress [50]. The PPARγ pathway was one mechanism of triclosan-induced mitochondria-targeted effects, regulating the function of these organelles and the permeability of their membranes [51,52]. Our research showed that PPARγ activation or overexpression mitigated, while PPARγ inhibition or silence aggravated the increase of inflammatory gene expression caused by TCS. It is interesting that PPARγ prevented TCS-induced toxic phenotypes, while treatments with a PPARγ agonist or antagonist alone had no effects, suggesting some new mechanisms related to PPARγ can be initiated by TCS, which is worth more future study.

**Figure 8:**
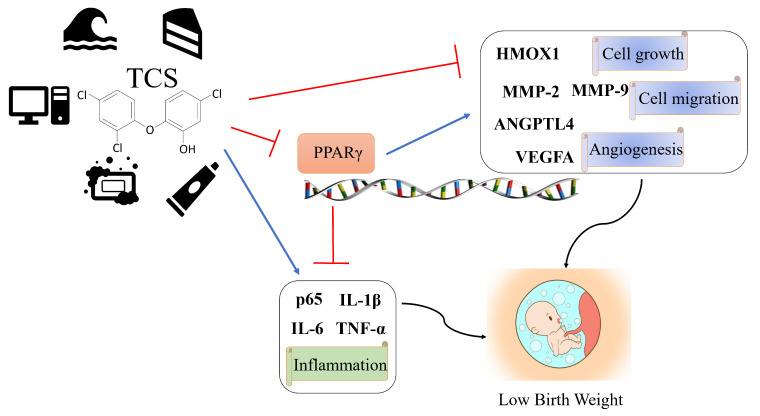
Schematic model depicting TCS-induced placental dysfunction and low birth-weight infants through PPARγ signaling pathways. TCS inhibited cell growth, angiogenesis, and migration and promoted inflammation of placenta via PPARγ-regulated genes *HMOX1*, *ANGPTL4*, *VEGFA*, *MMP-2*, *MMP*-9, *p65*, *IL-6*, *IL-1β*, and *TNF-α*.

All the results in this study illustrated that TCS caused significant abnormal functions of HTR-8/SVneo and JEG-3 cells, including viability, migration, angiogenesis, and inflammation response. In addition, treatment with rosiglitazone or overexpression of PPARγ almost completely prevented the abnormal cell function changes in vitro. On the other hand, treatment with GW9662 or *si-PPARγ* aggravated the toxicity. Similarly, animal studies also indicated that rosiglitazone mitigated the decrease of placental diameter and fetal weight caused by TCS. In addition, curcumin, a natural compound and a modulator of PPARγ, has been known to have beneficial effects on pregnancy outcomes [53,54]. It will be interesting to know the effect of curcumin or other natural PPARγ modulators on TCS placental exposure in the future. Our results suggested that TCS placental exposure had adverse effects in vitro and in vivo through the PPARγ pathway (Figure 8), and activation or increased expression of PPARγ is a potential strategy to protect against the placenta toxicity induced by TCS.

## Data Availability

The data sets generated and/or analyzed during the current study are available from the corresponding author on reasonable request.

## Supplementary Materials

The following supporting information can be downloaded at: https://www.mdpi.com/article/10.3390/cells11010086/s1, Figure S1: The efficiency of PPARγ overexpression and knockdown; Table S1: RT-PCR primers.
