# Supplementary material for: PPARγ Regulates Triclosan Induced Placental Dysfunction
Source: Cells. 2021 Dec 28;11(1):86. doi: 10.3390/cells11010086 (PMC8750171; doi:10.3390/cells11010086)
Supplement: Supplementary file 1 [file cells-11-00086-s001.zip › cells-1478137-supplementary.pdf]

Jing Li et al. PPARγ Regulates Triclosan Induced Placental Dysfunction

Supplementary Figure S1.

Figure S1

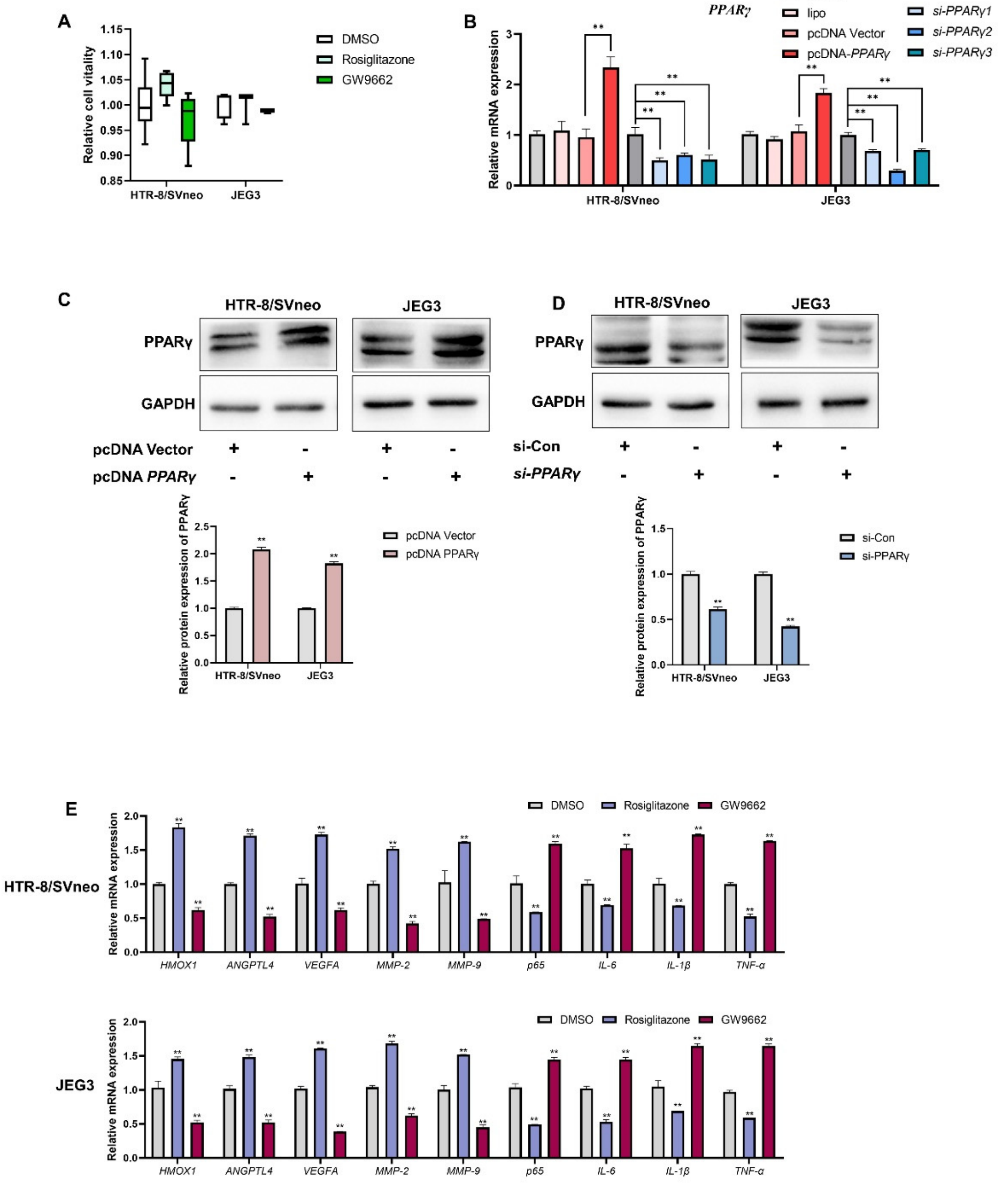

Supplementary Figure S1. (A) Cell vitalities were detected by CCK-8 assay when exposed to rosiglitazone and GW9662 in HTR-8/SVneo and JEG-3 cells. (B) PPAR-γ-pcDNA and si-PPARγ were transfected into cells and transfection efficiency were detected by RT-PCR. RNA was isolated from cells and then RT-PCR was used to evaluate the transcription of PPARγ. pcDNA-PPARγ (C) and si-PPARγ (D) were

transfected into cells and transfection efficiency were detected by western blot. Protein was isolated from cells and then western blot was used to evaluate the transcription of PPAR-γ. (E) The mRNA expression of PPARγ regulated genes were analyzed by RT-PCR in the absence or presence of rosiglitazone or GW9662 for 24 h in HTR-8/SVneo and JEG-3 cells. The data are shown as the means ± S.E.M. *$P < 0.05$; ** $P < 0.01$; compared with the indicated group, n=3.

Supplementary Table S1. **RT-PCR primers**

Gene symbol	Forward primer	Reverse primer
hGAPDH	GGAAATCCCATCACCATCT	GGACTCCACGACGTACTCA
hANGPTL4	CTCAAGGCTCAGAACAGCAGG	TGGTCCAGGAGGCCAAACT
hHMOX1	CAGCGGGCCAGCAACAAAG	ACCCATCGGAGAAGCGGAGC
hVEGFA	AGGGCAGAATCATCACGAAG	GAAGATGTCCACCAGGGTCTC
hMMP-2	CTTCCAGGGCACATCCTAT	CCTTCTGAGTTCCCACCAA
hMMP-9	TCCCTGGAGACCTGAGAACC	GCCACCCGAGTGTAACCAT
hp65	GGGGACTACGACCTGAATGCT	GTCAAAGATGGGATGAGAAAGGA
hTNF-α	TGAAAGCATGATCCGGGACG	AGGCAGAAGAGCGTGGTGGC
hIL-6	CAAATTCGGTACATCCTCG	TTTCTGCCAGTGCCTCTTT
hIL-1β	ATGGCTTATTACAGTGGCA	GTAGTGGTGGTCGGAGATT
mGapdh	TCTTGGGCTACACTGAGGA	ATACCAGGAAATGAGCTTGA
mAngptl4	ATCACAGGGAACCGAGGAA	ATTGGAGCAATTTGGCATT
mHmox1	GGTGATGGCTTCCTTGTAC	AGACTGGGTTCTGCTTGTT
mVegfa	GCACCCACGACAGAAGGAG	TCAATCGGACGGCAGTAGC
mMmp-2	TGTCCCGAGACCGCTATGT	TTGCCCAGGAAAGTGAAGG
mMmp-9	ACAGCCAACTATGACCAGGAT	TTGCCCAGGAAGACGAAGG
mp65	TGCGATTCCGCTATAAATG	TTGGTGGTATCTGTGCTTCTC
mTnf-α	TCTCATTCCTGCTTGTGGC	GGAACTTCTCATCCCTTTGG
mIl-6	CTTCTTGGGACTGATGCTG	GGTCTGTTGGGAGTGGTAT
mIl-1β	TGAAGGGCTGCTTCCAAAC	GATGTGCTGCTGCGAGATT
